# Effect of Early Post Cesarean Feeding on Gastrointestinal Complications

**DOI:** 10.5812/nms.10184

**Published:** 2013-06-27

**Authors:** Mohadese Adeli, Nastaran Razmjoo, Fatemeh Tara, Saeed Ebrahimzade

**Affiliations:** 1 Torbateheydariyeh University of Medical Sciences, Torbateheydariyeh, IR Iran; 2 Department of Midwifery, Faculty of Nursing & Midwifery, Mashhad University of Medical Sciences, Mashhad, IR Iran; 3 Department of Obstetrics & Gynecology, School of Medicine, Mashhad University of Medical Sciences, Mashhad, IR Iran; 4 Department of Biostatistics, Faculty of Nursing & Midwifery, Mashhad University of Medical Sciences, Mashhad, IR Iran

**Keywords:** Early-feeding, Cesarean section, Gastrointestinal

## Abstract

**Background:**

Gastrointestinal complications are the main complication in patients after cesarean section. Previous studies have reported different results about the effect of early post cesarean feeding on vomiting, nausea, flatulence and illus.

**Objectives:**

To identify the effect of early post cesarean feeding on gastrointestinal complications.

**Materials and Methods:**

This randomized controlled trial was conducted on 82 women who underwent cesarean section in Mashhad Omolbanin hospital. They were randomly assigned to two equal experimental and control groups. The experimental group started oral fluids four hours after surgery, followed by a regular diet after bowel sounds returned. Mothers in the control group received fluid intravenously during the initial 12 hours, and then if bowel sounds were heard, they were permitted to receive oral fluids and they could start a solid diet if they had defecation. Vomiting and flatulence were assessed with a visual analog scale. Nausea was assessed with an observation questionnaire and illus was assessed via bowel sounds, gas passing and defecation 4, 12, 24, 36 and 48, hours post surgery in the two groups. Also, they were studied for the time of gas passing, bowel sound return, defecation, sitting, walking and breast-feeding. Data were analyzed using the chi-square, Fisher's exact test, t-test and Man-Whitney U test.

**Results:**

No mother experienced nausea, vomiting and illus. Flatulence severity 4 and 12 hours after surgery was similar in both groups (P = 0.856, P = 0.392). However, flatulence severity 24, 36 and 48 hours after surgery, was less in the experimental group (P = 0.030, P = 0.016, P = 0.001). Also, bowel sound return, time of gas passing, defecation, sitting and walking were less in the experimental group (P = 0.001).

**Conclusion:**

This study showed that early feeding decreased post cesarean gastrointestinal complications.

## 1. Background

The number of cesarean sections performed each year is increasing at a dramatic rate all around the world. Therefore, postoperative care of these women demands attention. One of the general postoperative complications is gastrointestinal problems (1). Ileus (2-4), flatulence (5, 6) nauseas and vomiting (7, 8) are the most important problems after cesarean section leading to mother's dissatisfaction and prolong hospitalization (9, 10). Postoperative ileus cause intestinal gas retention, abdominal distension, nausea and abdominal pain (4). Ileus occur as a result of peristalsis decrease, manipulation of intestine and immobility (11). Prevention and reduction of gastrointestinal complications after cesarean section should follow the safest and most inexpensive method (6). Studies have considered post-operative feeding time as one of the factors affecting gastrointestinal complications. Although, some studies have confirmed post operative early-feeding, many hospitals still stick to their traditional approach (7). Traditionally, postoperative feeding following cesarean section involved consumption of 2-3 liters of intravenous fluids in the first 12-24 hours and oral intake is usually allowed after 24 hours in the absence of nausea and presence of detectable bowel activity. Regular diet is initiated after flatus is passed to avoid nausea, vomiting and abdominal distention (7, 12).

Nowadays, most researchers believe that after a nonevent cesarean section, patients could receive oral fluids as soon as they have recovered from anesthesia and experienced thirsty, and they can start a solid diet in a far shorter time-span than the traditional method (6, 13).

Abd Rabbo reported that early oral fluid consumption and post-operative early feeding cause belching by which flatulence and abdominal distension are relived due to an upward gas pass through the esophagus and stomach and removal of gas via the mouth. Post-operative early solid diet leads to less flatulence and results in a faster peristaltic return and also prevents gas retention in the colon (14). Shamaeian Razavi confirmed that early oral fluid intake decreased flatulence on the second and third day post-operation (6). On the other hand, some of the studies showed that early-feeding had no effect on flatulence and abdominal distension (4, 5, 7, 15, 16). One study also showed that early solid diet could increase post operative nausea and vomiting (8). As, there are different results from various studies in terms of the effect of post operative early-feeding on gastrointestinal complications, and since in most studies post cesarean feeding was not initiated earlier than six hours after surgery, the question raised is whether the feeding time of less than six hours can be associated with gastrointestinal complications. In other studies where post cesarean feeding began earlier than eight hours, the experimental group started liquid and solid diets without paying attention to the return of bowel sound during the initial eight hours after surgery.

## 2. Objectives

To identify whether the kind of diet (liquid and solid) is effective on gastrointestinal complications.

## 3. Materials and methods

This randomized controlled trial was conducted on 82 women who underwent cesarean section in Mashhad Omolbanin hospital during 2009. These patients were allocated into two groups (41 patients in each group). (the sample size was calculated based on a study in which the mean standard deviation of flatulence severity was 43.44 ± 24.91 and 57.73 ± 19.60 in the two groups. (The type I error probability and the power of the study were selected to be is 0.05 and 0.80 respectively).

Mothers were selected consecutively if they had the inclusion criteria and assigned randomly into the two equal groups. Inclusion criteria included, literacy (ability to read and write), having a single pregnancy, gestational age between 38 and 42 weeks, having a normal birth with first-minute APGAR score of 7 - 10, having an elective cesarean section with transverse incision and general anesthesia (maximum of one previous cesarean), having normal vital signs, do not using of laxative (anti flatulence) food or drugs at home, and not having a history of bowel surgery, smoking and addiction. Exclusion criteria were: fetal anomaly, medical or obstetric complications, intera-operative or immediate major postoperative complications (such as the need for blood transfusion for any reason, intolerance to oral liquid diet, postoperative fever, lack of mothers) and desire to withdraw from the study.

Data were collected using a questionnaire, interview and observation checklist . These tools consisted of questions about demographic characteristics, reproductive data and information about the patient before, during and after the cesarean section.

A visual analog scale (VAS) was used to assess vomiting and flatulence. The scale consists of a horizontal line 100 mm in length, which was scaled between 0 and 100. We presented this scale to the mother and asked her to identify her severity of vomiting and flatulence by marking this line. The variables were then measured by calculating the distance from the zero point.

Immediately after surgery, patients were randomized into two groups: the early-fed group and the traditional group. The early-feeding mothers received IV fluid just four hours after surgery (120 mL/h) and IV therapy was discontinued; these mothers could receive 30 mL (a half cap) of diluted sweat fluids including water and tea sweated by sugar and candy, four hours after surgery. If they could tolerate, this amount would be doubled and they could receive 60 mL (one cup) of fluid and this was increased based on individuals' need accordingly. If there was no nausea and vomiting and they could tolerate fluid, they could start clear fluids such as soup, fruit juice, milk and yoghurt regardless of bowel sounds return, so that they could consume 1500 cc/24h. They could start a regular diet as soon as bowel sounds were heard. For the traditional feeding group, the routine method of post surgical care was implemented i.e. mothers received 1.5 liter of fluid intravenously during the initial 12 hours, then if bowel sounds were heard, they were permitted to receive a diluted clear fluid diet such as water, fruit juice, tea, soup, milk and yoghurt and they could start a solid diet if they had defecation.

IV line and urinary catheter were discontinued for both groups after 12 h postoperatively. The volume of urine was recorded every four hours by graded measures for 24 h. The two groups received narcotics and antibiotics based on the hospital routine. They were controlled for nausea times and severity, vomiting and flatulence severity during 4, 12, 24, 36 and 48 h after surgery with a VAS. Bowel sounds were controlled every 30 minutes and the first gas passing and defecation time were recorded. Total IV and oral fluid intake in the first 24 hours, start of regular diet, first sitting and walking time and breastfeeding time were also recorded during the first 24 h. All mothers had a daily visit by the gynecologist.

### 3. 1. Ethical considerations

University ethics committee’s approval was obtained and all subjects gave prior written informed consent. As part of the informed consent process, patients were advised that they had an equal chance of being randomized into the two groups of either the early feeds or the traditional feeds. How early the feeds were, was not specified, to keep patients blinded to their group.

### 3. 2. Data analysis

At first, Kolmogroff Smirnov test was used to determine the normality of all variables. To compare the two groups of quantitative variables with normal distribution, t-test was used and in cases of non-normal distribution Mann-Whitney U test was used. Also chi-square test and Fisher's exact test were used. In all cases the confidence interval was calculated. A P value less than 0.05 was considered as significant in all tests.

## 4. Results

The mean age of mothers was 25.6 years. 91.5% of mothers were housewives and 35.4% of them had high school education. Most of them were satisfied with their economic status (74.4%), 26.8% had one previous abortion and 7.3% of them reported a previous dead fetus. According to the results, both groups were similar in terms of age (P = 0.327), education level (P = 0.088), spouse education (P = 0.480), spouse job (P = 0.354), economical satisfaction (P = 0.077), planned pregnancy (P = 0.820), satisfaction about the infant’s gender (P = 1.000), previous abortion (P = 0.618), previous dead fetus (P = 0.494), pregnancy care (P = 0.159), gestational age (P = 0.189), duration of pre-operative hospitalization (P = 0.193), preoperative fasting (P = 0.054), anesthesia length (P = 1.000), surgery length (P = 0.146), pre-medication (P = 0.696), birth weight (P = 0.901), first (P = 0.308) and fifth minute APGAR Score (P = 0.535) and infant gender (P = 0.267).

As shown in table 1, there was no significant difference between the groups with respect to the preoperative and intraoperative information.

**Table 1.** Comparison of Preoperative and Intraoperative Information

	Early-feeding, (n = 41)	Traditional feeding, (n = 41)	P Value
**Planned pregnancy, No. (%**)	26 (63.4)	25 (61.0)	0.820^a^
**Satisfaction about Infant’s gender, No. (%)**	41 (100)	40 (97.6)	1.000^a^
**Previous abortion, No. (%)**	10 (24.4)	12 (29.3)	0.618^a^
**Previous dead fetus, No. (%)**	2 (4.9)	0 (0)	0.494^a^
**Gestational age at delivery, week, Mean ± SD**	39.0 ± 0.8	39.3 ± 1.0	0.189^b^
**Gravity, Mean ± SD**	2.3 ± 0.6	2.4 ± 0.6	0.822^c^
**Parity, Mean ± SD**	2.0 ± 0.0	2.1 ± 0.3	0.079^c^
**Preoperative hospital stay,hr, Mean ± SD**	12.9 ± 1.5	13.3 ± 1.5	0.193^c^
**Preoperative NPO,hr, Mean ± SD**	11.3 ± 1.3	11.9 ± 1.1	0.054^c^
**Anesthesia length, Min, Mean ± SD**	59.8 ± 1.1	59.8 ± 1.1	1.000^c^
**Surgery length, Min, Mean ± SD**	49.63 ± 2.6	48.78 ± 2.7	0.146^c^

^a^ chi- square test

^b^ t-test

^c^ Mann- Whitney test

No mother experienced nausea, vomiting and lleus. Postoperative flatulence severity 4 and 12 hours after surgery was similar in both groups (P = 0.856 and P = 0.392 respectively). However, flatulence severity 24, 36 and 48 hours after surgery, was less in the experimental group (P = 0.030, P = 0.016, and P = 0.001 respectively). Also, time of hearing bowel sound, the first gas passing time and the first defecation were significantly less in the early- feeding group (P = 0.001) (Table 2).

**Table 2.** Comparing Gastrointestinal Complications in the Two Groups

Variable	Early-feeding, Mean ± SD	Traditional feeding, Mean ± SD	P Value
**Flatulence severity, 4h after cesarean**	3.0 ± 10.7	4.7 ± 12.2	0.856
**Flatulence severity, 12h after cesarean**	20.0 ± 20.7	19.1 ± 25.6	0.392
**Flatulence severity, 24h after cesarean**	13.9 ± 20.5	30.6 ± 32.9	0.030
**Flatulence severity, 36h after cesarean**	19.2 ± 28.1	35.1 ± 31.7	0.016
**Flatulence severity, 48h after cesarean**	6.5 ± 16.3	24.2 ± 25.6	0.001
**Time of hearing bowel sounds,hr^a^**	4.4 ± 0.7	6.7 ± 4.9	0.001
**The first gaspassing time,hr**	13.1 ± 4.6	19.8 ± 7.2	0.001
**The first defecation time,hr**	19.9 ± 5.9	24.6 ± 7.6	0.001

^a^ Abbreviation: hr; Hour

No significant difference was found between the two groups in fluid volume intake 4h after staring oral fluids (P = 0.898), while time of regular diet initiation in the early feeding group was less than the traditional group (P = 0.001) (Table 3).

**Table 3.** Comparing the Rate and Time of Receiving Fluids and Regular Diet in the Two Groups

Variable	Early-feeding, Mean ± SD	Traditional feeding, Mean ± SD	P Value
**Volume intake 4h after start of oral fluid, mL**	739 ± 220.9	745.1 ± 210.0	0.898^a^
**start of regular diet,hr**	8 ± 4.2	25.1 ± 7.8	0.001^b^

^a^ t-test

^b^ Mann-whitney test

The first sitting and walking time in the early-feeding group was significantly less than the traditional group (P = 0.001). The first breastfeeding time was not significantly different between the two groups (P = 0.678), while in the first 24 hours breastfeeding times were significantly higher in the early-feeding group (P = 0.037) (Table 4).

**Table 4.** Comparing Sitting, Walking and Breastfeeding Time in the Two Groups

Variable	Early-feeding, Mean ± SD	Traditional feeding, Mean ± SD	P value ^b^
**First sitting time, hr** ^a^	4.9 ± 1.3	12.4 ± 1.1	0.001
**First walking time,hr**	5.1 ± 3.2	12.5 ± 4.1	0.001
**First breastfeeding time, Min**	64.3 ± 32.3	73.5 ± 30.2	0.678
**First 24-hour breastfeeding time **	16.3 ± 6.1	13.1 ± 4.6	0.037

^b^ Mann-whitney test

^a^ Abbreviation: hr; hour, Min; Minutes

## 5. Discussion

Our study confirmed that early post cesarean feeding in women is well tolerated. There were no significant increases in the incidence of postoperative gastrointestinal complications in the early feeding group.

According to the results, no significant differences were found between the two groups in terms of nausea, vomiting and ileus. Although early feeding caused peristalsis acceleration, no significant difference was shown for flatulence severity four hours after operation. This is expected to be due to equal conditions of mothers and lack of oral intake. Flatulence severity 12 hours after operation was similar in both groups. Our results show that liquid and solid diets in the early feeding group had not increased flatulence. Flatulence severity 24 hours after operation was less than the traditional feeding group while Shamaeian Razavi ’s study reported that flatulence severity 24 hours after operation was similar in both groups. In Shamaeian Razavi ’s study, mothers in the study group received a solution of 50 gr glucose in 1000 ml of water Instead of IV fluid and then for initiation of oral fluids and a regular diet, the routine method of surgical ward was implemented (6). Thus, early feeding in their study was limited to a specific solution instead of IV fluid but in our study, the type of oral fluid and method were different. We believe that the difference in method and type of interval can effect the results. Also flatulence severity 36 and 48 hours after operation were less in the early feeding group.

It seems that flatulence severity in the early-feeding group was increased, 12 hours after operation due to a fluid and solid diet and then it was decreased 24 hours after surgery due to defecation of most mothers and once again it increased 36 hours after the operation, when mothers were discharged. It seems that receiving a voluminous meal for lunch at home led to this increase 36 hours post operatively, while in the traditional group, flatulence severity showed an increasing rate except 48h after surgery.

Shamaeian Razavi also emphasized that early oral fluid intake, decreased post-operative second and third day flatulence severity (6). Some of previous studies reported equal flatulence and abdominal distention in the two groups (4, 5, 7, 16). Our finding was in agreement with Shamaeian Razavi’s study, but it was not similar to that of Malhotra, Izbizky, Burrows and Kramer (5, 7, 16). It seems that these alternatives were caused by differences in methods because in the Malhotra report, the study group started a liquid diet during the first 6 hours after surgery, without specifying a time for everyone (7). Also lzbizki, Burrows and Kramer allowed the study group to receive a solid diet during the initial 8 hours after surgery without paying attention to the bowel sound return (4, 5, 16).

In our study none of the mothers were excluded due to a lack of desire for drinking. Start of a regular diet in the early feeding group was shorter because the first defecation time in the early-feeding group was less than the traditional group. Their flatulence was less so mothers tended to eat sooner. Shamaeian Razavi, Malhotra and Teoh et al., also concluded that early fluid diet caused early regular diet and solid diet tolerance in a shorter period (6-8). Statistical significant difference was found between the two groups for bowel sounds hearing time, first gas pass and first defecation time. Several studies also confirmed that post cesarean early oral intake caused faster bowel sounds return (10, 13, 16, 17). However, Burrows (16) stated that early feeding has no effect on bowel sounds returning time and lzbiki (5) also reported no significant difference for hearing bowel sounds. Our findings are similar to that of some of previous studies (3, 6, 7, 9, 10, 17) but they are in disagreement with some others (5, 16). In the Izbiki and Burrows study (5, 16), experimental group started solid diet without hearing bowel sounds during the first 8 hours after surgery, while in the present study, the early feeding-group received clear fluid without hearing bowel sounds and they could start a regular diet after hearing bowel sounds. This showed that a fluid diet is more stimulant than a solid diet for bowel sounds return.

According to the result, post operative early-feeding, not only did not increase gastrointestinal complications, but also, it could be well tolerated and led to advantages such as flatulence decrease, earlier bowel sounds return, earlier mothers' movement, earlier IV fluid discontinuation, successful breastfeeding and maternal-neonatal emotional development. 

Limitations of this study consist of personal, cultural and psychological differences between mothers for expression of flatulence and vomiting severity. All of mothers were discharged 24 hours after surgery (according to the hospital routine) so the VAS scale 36 and 48 hours after surgery was completed at home but researchers had adequate supervision. Also we asked mothers to stop using food or drug laxatives at home, however, the confidence in mothers to do so, is a limitation of this study. Thus we suggest further investigations without these limitations.

## References

[1] Fazel N, Tafazoli M, Ramezani M, Esmaili H (2005). [The effect of superminet on post cesarean flatulence].. Res Sci J Ardabil Univ Med Sci Health Serv..

[2] Chantarasorn V, Tannirandorn Y (2006). A comparative study of early postoperative feeding versus conventional feeding for patients undergoing cesarean section; a randomized controlled trial.. J Med Assoc Thai..

[3] Gocmen A, Gocmen M, Saraoglu M (2002). Early post-operative feeding after caesarean delivery.. J Int Med Res..

[4] Kramer RL, Van Someren JK, Qualls CR, Curet LB (1996). Postoperative management of cesarean patients: the effect of immediate feeding on the incidence of ileus.. Obstet Gynecol..

[5] Izbizky GH, Minig L, Sebastiani MA, Otano L (2008). The effect of early versus delayed postcaesarean feeding on women's satisfaction: a randomised controlled trial.. BJOG..

[6] Shamaeian Razavi N (2000). [The effect of post cesarean early oral fluid on postoperative gasterointestioneal compliments].. J Gonabad Univ Med Sci Health Serv..

[7] Malhotra N, Khanna S, Pasrija S, Jain M, Agarwala RB (2005). Early oral hydration and its impact on bowel activity after elective caesarean section--our experience.. Eur J Obstet Gynecol Reprod Biol..

[8] Teoh WH, Shah MK, Mah CL (2007). A randomised controlled trial on beneficial effects of early feeding post-Caesarean delivery under regional anaesthesia.. Singapore Med J..

[9] Bar G, Sheiner E, Lezerovizt A, Lazer T, Hallak M (2008). Early maternal feeding following caesarean delivery: a prospective randomised study.. Acta Obstet Gynecol Scand..

[10] Mulayim B, Celik NY, Kaya S, Yanik FF (2008). Early oral hydration after cesarean delivery performed under regional anesthesia.. Int J Gynaecol Obstet..

[11] Potter P, Pery A (2007). Principles and techniques of nursing. Translation: faculty members of Iran University of. Medical Science.

[12] Horowitz IR, Thompson JD, Rock JA, Horowitz IR, Thompson JD, Rock JA (1997). Postanaesthesia and postoperative care.. Telinde s operative gynecology..

[13] Kovavisarach E, Atthakorn M (2005). Early versus delayed oral feeding after cesarean delivery.. Int J Gynaecol Obstet..

[14] Abd Rabbo S (1995). Early oral hydration: a novel regimen for management after elective cesarean section.. J Obstet Gynaecol (Tokyo 1995)..

[15] Burrows WR, Gingo AJ, Jr, Rose SM, Zwick SI, Kosty DL, Dierker LJ, Jr (1995). Safety and efficacy of early postoperative solid food consumption after cesarean section.. J Reprod Med..

[16] Shafei A (2004). The tolerance of fluid diet at 6 and 12 hours after cesarean section.. Gorgan Med Univ J..

[17] Adupa D, Wandabwa J, Kiondo P (2003). A randomised controlled trial of early initiation of oral feeding after caesarean delivery in Mulago Hospital.. East Afr Med J..

